# Beneficial Effects of Maprotiline in a Murine Model of Colitis in Normal and Reserpinised Depressed Rats

**DOI:** 10.1155/2014/359841

**Published:** 2014-11-13

**Authors:** Mohsen Minaiyan, Valiollah Hajhashemi, Mohammad Rabbani, Ehsan Fattahian, Parvin Mahzouni

**Affiliations:** ^1^Department of Pharmacology and Isfahan Pharmaceutical Sciences Research Center, School of Pharmacy and Pharmaceutical Sciences, Isfahan University of Medical Sciences, Isfahan 814673461, Iran; ^2^Department of Pharmacology, School of Medicine, Shahrekord University of Medical Sciences, Shahrekord 8815774667, Iran; ^3^Department of Clinical Pathology, School of Medicine, Isfahan University of Medical Sciences, Isfahan 814673461, Iran

## Abstract

*Background*. Anti-inflammatory and immunomodulatory activities have been reported for maprotiline, a strong norepinephrine reuptake inhibitor. In addition, some other antidepressant drugs have shown beneficial effects in experimental colitis. *Methods*. All the animals were divided into normal and depressed groups. In normal rats colitis was induced by instillation of 2 mL of 4% acetic acid and after 2 hours, maprotiline (10, 20, and 40 mg/kg, i.p.) was administered. In reserpinised depressed rats, depression was induced by injection of reserpine (6 mg/kg, i.p.), 1 h prior to colitis induction, and then treated with maprotiline (10, 20, and 40 mg/kg). Treatment continued daily for four days. Dexamethasone (1 mg/kg, i.p.) was given as a reference drug. On day five following colitis induction, animals were euthanized and distal colons were assessed macroscopically, histologically, and biochemically (assessment of myeloperoxidase activity). *Results*. Maprotiline significantly improved macroscopic and histologic scores and diminished myeloperoxidase activity in both normal and depressed rats while reserpine exacerbated the colonic damage. *Conclusion*. Our data suggests that the salutary effects of maprotiline on acetic acid colitis are probably mediated first through depressive behavioral changes that could be mediated through the brain-gut axis and second for the anti-inflammatory effect of the drug.

## 1. Introduction

The inflammatory bowel diseases (IBD), such as Crohn's disease and ulcerative colitis (UC), affect approximately 1-2 of every 1000 people in developed countries [1]. They are chronic and relapsing inflammatory disorders of the gastrointestinal tract defined by clinical characteristics such as diarrhea, abdominal pains, weight loss, and nausea and by pathological features such as loss of mucosal integrity and inflammatory cell infiltration [2]. These chronic diseases result in serious complications and continue to be a common cause of morbidity that aggravates the quality of life. Anxiety and depression are also known to independently affect quality of life and may additionally impair quality of life in IBD over and above the IBD itself [3, 4].

Depression and anxiety are significantly more common among people with IBD than in control population [5–9]. The prevalence of anxiety and/or depression has been estimated to be as high as 29–35% during remission and 80% for anxiety and 60% for depression during relapses [10]. The presence of clinical depression in patients with IBD may influence disease activity, aggravate disease symptoms, impact on the evolution of the disease, and may affect their response to standard treatment for IBD [11]. Some researchers have further proposed that anxiety and depression may influence the clinical course of IBD. They have studied and expressed the contribution of the psychological disorders to the inflammatory process in inflammatory bowel disease (IBD) as contribution to disease onset, to worsening of inflammation and failure of treatment response, and to relapse of inflammation [12]. Recent studies have provided strong evidence for plausible mechanisms by which stress effects could be transduced into gut inflammation, including stress-induced changes in intestinal permeability which result in reducing mucosal barrier function and/or stress-induced changes in mucosal proinflammatory cytokines which result in exacerbating the immune dysfunction [13].

Although there are some conflicting results, it has been suggested that most of the antidepressant drugs, such as amitriptyline, nortriptyline, imipramine, and doxepin, in addition to their use for the management of depression, are broadly used to alleviate various types of pain such as inflammatory (rheumatoid arthritis) pain [14–16]. Antidepressants can also lower the levels of systemic inflammation markers, such as C-reactive protein and cytokines in many inflammatory conditions [17]. Although various classes of antidepressants are available for physicians to prescribe, it is clear from preclinical and clinical data that antidepressant drugs have no equal anti-inflammatory effect. There are several reports about the analgesic and anti-inflammatory effects of antidepressant drugs; for instance, anti-inflammatory activity of fluoxetine, a selective serotonin reuptake inhibitor (SSRI), was studied on the carrageenan-induced paw inflammation in the rat [18]. Antinociceptive and anti-inflammatory effects of venlafaxine in the rat model of inflammation were evaluated and it is shown that pretreatment with venlafaxine significantly reduced or completely abolished the enhanced sensitivity to mechanical stimuli provoked by peripheral carrageenan injection [19]. Additionally anti-inflammatory effect of amitriptyline [20], fluvoxamine [21], and maprotiline [22] on carrageenan-induced paw edema has been evaluated in our laboratory. Maprotiline is a strong norepinephrine reuptake inhibitor with weak effects on serotonin and dopamine reuptake. This drug is well tolerated with fewer side effects and less interference with autonomic system compared with first generation antidepressants [23]. In a recent study it is shown that both i.p. and i.c.v. maprotiline considerably decreased paw edema four hours after subplantar injection of carrageenan [21].

So the present study was designed to investigate the beneficial effects of maprotiline, a tetracyclic antidepressant, against acetic acid-induced colitis in normal and reserpinised depressed rats.

## 2. Materials and Methods

### 2.1. Chemicals

The maprotiline was a gift from Razak Pharmaceutical Company (Tehran, Iran). Dexamethasone was also a gift from Raha Pharmaceutical Company (Isfahan, Iran). Reserpine, hexadecyltrimethyl-ammonium bromide (HTAB), and* o*-dianisidinedihydrochloride were purchased from Sigma Chemical Co. (St. Louis, Mo, USA). Formalin solution 35% w/w, glacial acetic acid, and diethyl ether were purchased form Merck (Darmstadt, Germany). All other solvents and chemicals were of analytical grade.

### 2.2. Animals

Male Wistar rats (200–250 g) were obtained from the animal house of the School of Pharmacy, Isfahan University of Medical Sciences, Iran. Rats were fasted for 24 h before induction of colitis in stainless steel cages with free access to water. During experiment, the animals were housed in standard polypropylene cages, four per cage, under a 12:12 h light/dark cycle with free access to food and water. The experiments were carried out in accordance with local guidelines for the care of laboratory animals of the Isfahan University of Medical Sciences.

### 2.3. Behavioral Tests

#### 2.3.1. Determination of Antidepressant Dose of Maprotiline in Reserpinised Depressed Rats

Thirty-six rats were randomly divided into the following groups of six rats in each. Sham group received intraperitoneally (i.p.) injection of normal saline daily for four days; control group received reserpine (6 mg/kg, i.p.) at the first day and daily normal saline for four days; and test groups received reserpine (6 mg/kg, i.p.) at the first day and daily maprotiline (5, 10, 20, and 40 mg/kg, i.p.) for four days.

At the third day, the rats were individually placed in a cylinder containing water 15 cm in height at 25°C for 15 min. On the following day (fourth day) the rats were again immersed in water and total duration of immobility was measured for 5 min. The immobility time was regarded as the time the mouse spent floating in the water without struggling and making only those movements necessary to keep its head above water (forced swimming test) [24].

### 2.4. Induction of Experimental Colitis

Distal colitis was induced by intracolonic instillation of 2 mL of acetic acid (4% v/v in 0.9% saline) according to MacPherson and Pfeiffer's method [25]. After 24 h fasting, each rat was lightly anesthetized with ether and a polypropylene catheter was inserted into the colon via the anus. The catheter was advanced so that the tip was 8 cm proximal to the anus. At this point acetic acid was instilled. Then the rat was kept in a head-down position for 30 s to limit expulsion of the solution.

### 2.5. Experimental Groups

Animals were randomly divided into the following groups each consisting of six. Sham group underwent the cannulation procedure without colitis induction, receiving normal saline instead of acetic acid. Control group received normal saline (1 mL/kg, i.p.), respectively, after induction of colitis. Test groups included nonreserpine treated groups which received maprotiline (10, 20, and 40 mg/kg, i.p.) and reserpine treated groups (6 mg/kg, i.p.), 1 h prior to induction of colitis and then treated with maprotiline (10, 20, and 40 mg/kg, i.p.), and reference group which received dexamethasone (1 mg/kg, i.p.) after induction of colitis.

All treatments were carried out 2 h after colitis induction and continued daily for four days.

### 2.6. Measurement of Body Weight Changes

Body weight was recorded for each animal during the experimental period (prior to induction of colitis and subsequently daily over the test period).

### 2.7. Evaluation of Colon Macroscopic Damage

Rats were euthanized by an overdose of ether anesthesia at the fifth day (the day after receiving the last dose). Abdomen was opened and colon was exposed. Distal colon, 8 cm in length and 2 cm proximal to the anus, was excised and opened longitudinally. The tissue of colon was rinsed with normal saline and wet weight was measured [26]. Then, the tissue was fixed on a white plastic sheet and a photo was taken using an appropriately adjusted Nikon camera (Coolpix p100). Macroscopic damage scores were assigned by an independent observer according to the following criteria: 0 = no macroscopic changes, 1 = mucosal erythema only, 2 = mild mucosal edema, slight bleeding, or slight erosion, 3 = moderate edema, bleeding ulcers, or erosions, and 4 = severe ulceration, erosions, edema, and tissue necrosis [27]. Furthermore, ulcer area was measured by Fiji-win 32 software, an image processing and analysis software inspired by NIH image for the Macintosh [28]. For each specimen ulcer index was calculated using the following equation [29]:

(1) $$\begin{array}{c} \text{Ulcer}\;\;\text{Index}=\text{Ulcer}\;\;\text{area}\;\;\left({\text{Cm}}^{2}\right)+\text{Macroscopic}\;\;\text{score}. \end{array}$$

After that, the colon specimen was bisected in the middle equally. One part was frozen in liquid nitrogen and kept at freezer (−74°C) in order to measure the myeloperoxidase (MPO) enzyme activity. Another part was fixed in 10% formalin solution in phosphate buffered saline for pathological examination [30].

### 2.8. Evaluation of Colon Histological Damage

To process for histopathological studies, colonic specimens which were fixed in 10% formalin in phosphate buffered saline embedded in paraffin and cut into 4 *μ*m sections. Then paraffin sections were deparaffinized with xylene and then hydrated and stained with hematoxylin and eosin (H&E). The stained sections were assessed for any inflammatory changes including infiltration of cells, necrosis, or damage to tissue structures [31]. Inflammation severity and extent as well as crypt damage were evaluated on H&E stained according to the criteria previously described by Dieleman et al. [32]. Total colitis index was measured by summing these three subscores (inflammation severity, inflammation extent, and crypt damage).

### 2.9. Myeloperoxidase Assay

Myeloperoxidase (MPO) enzyme activity, a marker of neutrophil infiltration, was measured in colon tissue according to the modified method of Bradley et al. [33]. The colon tissue was weighed and homogenized in 1 mL of 50 mM potassium phosphate solution (pH: 6) with 0.5% HTAB and 5 mM EDTA in an ice bath using polytron homogenizer. Additional buffer solution was added to obtain a concentration equivalent to 5 mL per 0.1 g of colon tissue. The resultant homogenate was sonicated in an ice bath for 10 s, then subjected to a sequence of freezing and thawing 3 times, and sonicated again for 10 s and centrifuged for 15 min at 15,000 rpm at 4°C. An aliquot of 0.1 mL of the supernatant was mixed with 2.9 mL of 50 mM phosphate buffer (pH: 6) containing 0.167 mg/mL* o*-dianisidinedihydrochloride and 0.0005% hydrogen peroxide. The change in absorbance at 460 nm was measured using a UV-Vis spectrophotometer (LSI Model, Alfa-1502).

### 2.10. Statistical Analysis

Results are expressed as means ± SEM for parametric and median (range) for nonparametric data and the minimum level of significance was considered at *P* < 0.05. All statistical analyses were assessed using GraphPad Prism 5 software. Differences among groups were tested by parametric one-way analysis of variance (ANOVA) with Tukey's HSD as post hoc test. Nonparametric data were analyzed using Kruskal-Wallis followed by Mann-Whitney* U* test.

## 3. Results

### 3.1. Behavioral Tests

#### 3.1.1. Assessment of Antidepressant Dose of Maprotiline Using Forced Swimming Test

**Figure 1:**
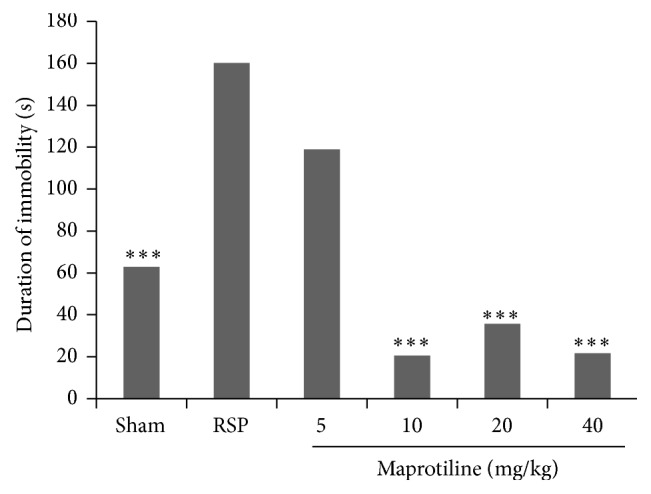
Effect of maprotiline (5, 10, 20, and 40 mg/kg, i.p.) on duration of immobility (seconds) during forced swimming test in reserpinised (6 mg/kg, i.p.) rats. RSP: reserpine (6 mg/kg); i.p.: intraperitoneally. Results are expressed as mean ± SEM of six rats in each group. ^***^ *P* < 0.001 compared to RSP; one-way ANOVA followed by Tukey's test.

As it is shown in Figure 1, maprotiline at doses of 10, 20, and 40 mg/kg significantly reduced the time spent immobile compared to reserpine group (*P* < 0.001). So these three doses of maprotiline were selected for evaluation in the ulcerative colitis rats.

### 3.2. Animals' Body Weight Loss

Twenty-four hours after the instillation of 4% acetic acid into the colon, the animals developed bloody diarrhea, weakness, and decreased food intake that resulted in body weight loss during the experiment period. Tables 1 and 2 summarize the data of body weight loss of Sham, control, and treatment groups.

As illustrated in Tables 1 and 2 control group showed loss of body weight on day five (*P* < 0.01) whereas i.p. injection of maprotiline reduced this body weight loss in nondepressed rats. In reserpine-induced (6 mg/kg, i.p.) depressed animals, control, reserpine (RSP, 6 mg/kg), and maprotiline (10 mg/kg) groups experienced a significant loss of body weight in comparison with Sham group. Additionally there is no significant difference between maprotiline at doses of 20, 40 mg/kg, dexamethasone, and Sham group in depressed and nondepressed rats.

### 3.3. Macroscopic Assessment

**Figure 2:**
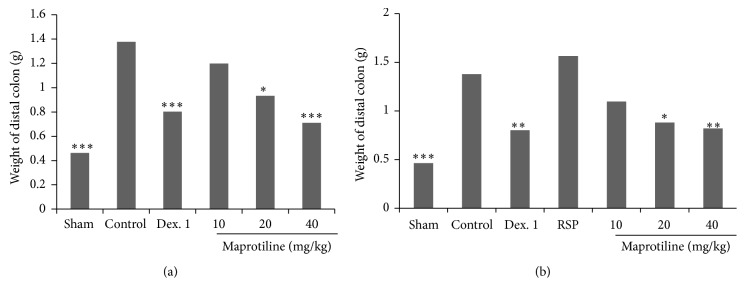
Effect of maprotiline (10, 20, and 40 mg/kg, i.p.) on weight of distal colon. (a) Normal rats and (b) reserpinised (6 mg/kg, i.p.) depressed rats. i.p.: intraperitoneally, Dex. 1: dexamethasone (1 mg/kg), and RSP: reserpine (6 mg/kg). Results are expressed as mean ± SEM of six rats in each group. ^*^ *P* < 0.05, ^**^ *P* < 0.01, and ^***^ *P* < 0.001 compared to control; one-way ANOVA followed by Tukey's test.

**Figure 3:**
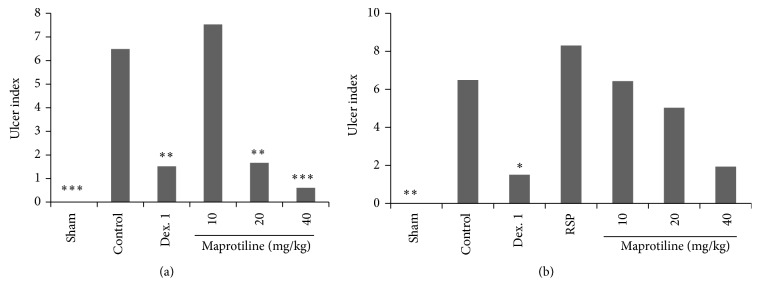
Effect of maprotiline (10, 20, and 40 mg/kg, i.p.) on ulcer index. (a) Normal rats and (b) reserpinised (6 mg/kg, i.p.) depressed rats. i.p.: intraperitoneally, Dex. 1: dexamethasone (1 mg/kg), and RSP: reserpine (6 mg/kg). Results are expressed as mean ± SEM of six rats in each group. ^*^ *P* < 0.05, ^**^ *P* < 0.01, and ^***^ *P* < 0.001 compared to control; one-way ANOVA followed by Tukey's test.

To assess the effect of maprotiline on macroscopic features in the rat model of colitis, two parameters including colon wet weight and ulcer index were evaluated in both normal and reserpinised (6 mg/kg, i.p.) depressed rats. In nondepressed animals, induction of colitis caused severe inflammation, ulceration, dilatation, adhesion, and wall thickening whereas colons of Sham group were normal (*P* < 0.001) (Figures 2 and 3).

In reserpinised depressed rats there was no significant difference between the reserpine (RSP, 6 mg/kg) group and control by considering both weight of colon and ulcer index (Figures 2(b) and 3(b)). So weight of colon tissue, ulcer index, and the severity of lesion with necrosis in group receiving reserpine (RSP, 6 mg/kg, i.p.) were increased. As expected, the reference drug, dexamethasone (1 mg/kg), caused a significant decrease in weight of colon and ulcer index in both normal and depressed rats (Figures 2 and 3). As it is shown in Figure 2, i.p. injection of maprotiline at doses of 20 mg/kg and 40 mg/kg significantly reduced the weight of colon in both normal and depressed rats.

As illustrated in Figure 3(a), i.p. injection of maprotiline at doses of 20 mg/kg (*P* < 0.01) and 40 mg/kg (*P* < 0.001) significantly and dose-dependently attenuated ulcer index as compared to control in nondepressed rats.

### 3.4. Assessment of Myeloperoxidase Activity

**Figure 4:**
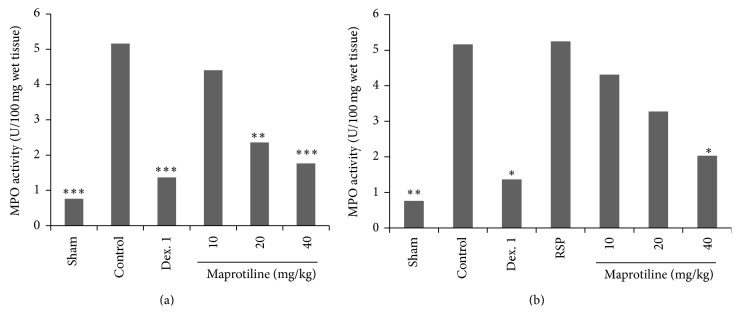
Effect of maprotiline (10, 20, and 40 mg/kg, i.p.) on myeloperoxidase (MPO) enzyme activity in the colonic tissue. (a) Normal rats and (b) reserpinised (6 mg/kg, i.p.) depressed rats. i.p.: intraperitoneally, Dex. 1: dexamethasone (1 mg/kg), and RSP: reserpine (6 mg/kg). Results are expressed as mean ± SEM of six rats in each group. ^*^ *P* < 0.05, ^**^ *P* < 0.01, and ^***^ *P* < 0.001 compared to control; one-way ANOVA followed by Tukey's test.

Colonic injury following acetic acid administration was accompanied by increased MPO activity, indicating neutrophil infiltration in inflamed tissue. As can be noted in Figure 4(b), there was a significant increase in colonic MPO activity in control and reserpine (RSP, 6 mg/kg) groups as compared to Sham. In reserpinised (6 mg/kg, i.p.) depressed rats, treatment with reserpine as a depressing agent significantly brought about a high MPO activity level same as that of control group. Conversely maprotiline treatment at dose of 20 mg/kg (*P* < 0.01) and 40 mg/kg (*P* < 0.001) and dexamethasone treatment (*P* < 0.001) significantly diminished the MPO activity level in nondepressed rat (Figure 4(a)). Additionally, in reserpine-induced depressed rats, maprotiline at dose of 40 mg/kg reduced the MPO activity level significantly (*P* < 0.05) (Figure 4(b)).

### 3.5. Pathological Assessment of the Colon

**Figure 5:**
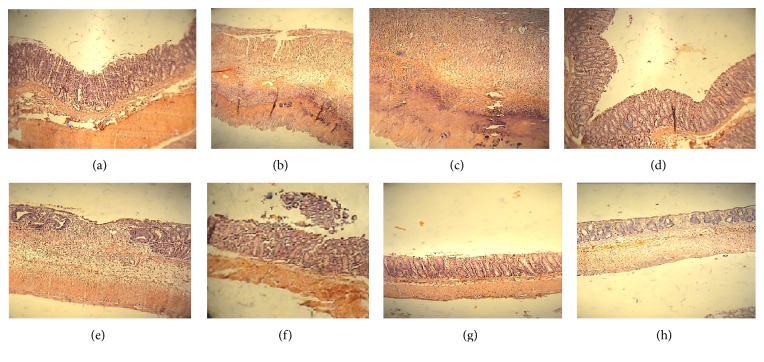
Histological appearances of colonic tissues in rats (H&E staining; magnification ×10). (a) Normal intact mucosa from Sham animals showed intact epithelial surface. (b) Colitis induced by acetic acid in control group: crypt damage, mucosal layers destruction, and leukocyte infiltration are evident. (c) acetic acid-induced colitis in reserpinised (6 mg/kg, i.p.) depressed rat, showing massive necrotic destruction of epithelium. (d, g, and h) Colitis tissue treated with dexamethasone (1 mg/kg, i.p.), maprotiline (20 mg/kg, i.p.), and maprotiline (40 mg/kg, i.p.) in nondepressed rats, respectively. (e, f) Colitis tissue treated with maprotiline (20 mg/kg, i.p.) and maprotiline (40 mg/kg, i.p.) in reserpinised (6 mg/kg, i.p.) depressed rats, showing attenuated the extent and severity of the histological signs of cell damage. i.p. = intraperitoneally.

Colonic mucosa of rats in Sham group had a normal architecture with intact epithelium (Figure 5). Pathologic assessment of colon in control group and reserpine (RSP, 6 mg/kg) group showed multifocal areas of necrosis, hemorrhage, submucosal edema, and inflammatory cell infiltration in lamina propria as well as loss of epithelium integrity (Figure 5). Administration of dexamethasone as reference and maprotiline at doses of 20, 40 mg/kg significantly attenuated the histopathological changes in both normal and reserpine-induced depressed rats (Table 3). Reepithelization of the mucosal layer and reduced inflammatory cell infiltration in propria were observed in these groups.

## 4. Discussion

The results of the present study clearly demonstrate that four days of administration of maprotiline, a tetracyclic antidepressant drug, at doses of 10, 20, and 40 mg/kg reduced the immobility time in forced swimming test in reserpinised (6 mg/kg, i.p.) depressed rats. Maprotiline also improved acetic acid-induced colitis in normal and reserpine-induced depressed rats. Administration of maprotiline as an antidepressant drug significantly decreased body weight loss and also increased body weight in normal rats. Biochemical and histological results also confirmed the effectiveness of maprotiline in improving acetic acid-induced colitis both in normal and reserpinised animals.

It is not deniable that people with inflammatory bowel disease (IBD) in general practice suffer from depression or anxiety as a reaction to living with this disease [34, 35]. On the other hand, it is believed that both depression and anxiety may also precede people with IBD through increasing permeability of colonic mucosa or increasing proinflammatory cytokines [36].

Despite the potential for anxiety and depression to play an important role in the clinical course of IBD, and despite the widespread antidepressant drugs, but according to available data, there is no any study to introduce a specific antidepressant drug which in addition to treating psychological disorders has beneficial effect in clinical course of IBD. So, for the first time, this paper reports the beneficial effect of maprotiline on experimental colitis in normal and reserpine-induced depressed rats.

It is evident that the immune system can fundamentally change in those suffering from major psychiatric disorders such as depression and anxiety disorders [37]. These mood disorders can stimulate production of proinflammatory cytokines and thereby adversely affect the course of IBD [36]. It is, therefore, a priority to pay careful attention to the possibility of mood disorders in patients with IBD. In the present study forced swimming test (FST) as a well-established animal test [24, 38] was used to evaluate reserpine-induced depression through increasing the duration of immobility time and also maprotiline by decreasing the duration of immobility time. This model is a valid model for evaluating depressive or antidepression effects [39, 40]. It is worth mentioning that maprotiline shows a strong antagonism against reserpine-induced effects in animal studies, as do the other classical antidepressants. Our result showed that reserpine (6 mg/kg, i.p.) significantly (*P* < 0.01) increased immobility time in FST as compared to control group and maprotiline (10, 20, and 40 mg/kg, i.p.) significantly (*P* < 0.001) antagonized reserpine-induced increase in mean immobility time in FST (Figure 1).

Acetic acid-induced colitis is an easily inducible model of IBD, and the similarity of the inflammatory mediators profile to IBD suggests that the inflammatory phase bears some resemblance to human intestinal inflammation. Intracolonic instillation of 4% acetic acid causes a relatively bland epithelial necrosis and edema that variably extended into the lamina propria, submucosa, or external muscle layers [41, 42]. In the present study dexamethasone is used as reference drug to compare the efficacy of treatment drugs on colitis and the results showed protection considering macroscopic and microscopic factors for applied drugs. In reserpinised depressed rats with no treatment (RSP group) we observed that colitis situation was exacerbated and epithelial necrosis and edema as well as body weight loss were exacerbated. But in groups which have received maprotiline both colitis status and body weight loss were improved.

There is a richly innervated nerve plexus between the enteric nervous system (ENS) and its spinal and autonomic connections to the central nervous system, known as the brain-gut axis. GI motor, sensory, and secretory functions as well as thresholds for the perception of pain can be affected by psychological disorders and emotional stress directly or indirectly through this axis. These effects are mediated by substance P (SP), vasoactive intestinal protein (VIP), several neuropeptides, neurotransmitters, and hormones [13]. In the present study depressive symptoms which are induced in reserpine-treated rats resulted in reduced food intake, weight loss, and exacerbation of macroscopic and histological features of colitis. Conversely improvement of the depressive symptoms by administration of maprotiline as an antidepressant possibly through the brain-gut axis improved food intake and other colitis parameters [43]. So maprotiline at doses of 20 and 40 mg/kg which showed antidepressant effect as confirmed by forced swimming test caused a dramatic reduction in the severity of colitis as indicated by improved macroscopic and histological features of IBD.

Reducing food intake with bloody diarrhea just twenty-four hours after induction of colitis is a convincing reason for weight loss in control group. Muscat et al. [44] reported that depression and chronic stress reduced the consumption of palatable sweet solution and weight loss such that this effect reversed by treatment with fluoxetine and maprotiline. As illustrated in Tables 1 and 2 depressions exacerbate body weight loss in reserpinised depressed rats and this effect reversed by administration of maprotiline.

During the inflammatory disease MPO content in the target tissue is increased and it is also augmented in both experimental and human IBD [45]. In this study, MPO activity in reserpine (RSP, 6 mg/kg) group as well as control group was markedly elevated showing that neutrophil infiltration to the inflamed tissue is increased following induction of colitis. Following five days treatment, maprotiline at doses of 20, 40 mg/kg diminished the elevated amounts of this biochemical marker in normal and depressed rats. According to pathological examination our result showed a marked reduction in the infiltration of PMN leucocytes into the inflamed colonic tissue compared to control (Table 3).

There is a bidirectional communication between neurons and mast cells within the gastrointestinal tract [36]. Dvorak and colleagues [46] reported that the number of mast cells was markedly increased in the involved area of the ileum of patients with IBD. Furthermore mucosal mast cells can be activated by stress and other psychological disorders. Activation of mast cells following neurological diseases such as depression, through release of mediators such as eicosanoids, serotonin, and IL-6, could contribute to the pathogenesis of IBD [47].

It has been shown that hypothalamus-pituitary-adrenal (HPA) axis function is reduced in patients with IBD; this observation may be relevant to stress induced increases in disease activity. Also, it has recently been demonstrated that reduced HPA axis function renders rodents susceptible to stress-induced increases in gastrointestinal inflammation [36]. In the present study maybe it is why depressed animal showed an increased susceptibility to acetic acid-induced colitis (reserpinised groups), an effect which was found to be dependent on coexisting stress.

It has been reported that anti-inflammatory effect of antidepressants is mediated through improving HPA function or inhibition of SP [18]. Ghia et al. reported that antidepressants can improve the autonomic dysfunction in IBD patients, thereby inhibiting proinflammatory mediator release [48]. So in the present study we hypothesized that maprotiline-induced alleviation of intestinal inflammation might be through reduction of these inflammatory mediators. Another possible mechanism is the direct stimulatory effect of maprotiline on cortisol secretion or indirect increase of this anti-inflammatory hormone via hypothalamic-pituitary-adrenal axis. However, reaching a definite mechanism needs further studies.

Antidepressants have been shown to inhibit the activation of gene expression of iNOS (inducible nitric oxide synthase) and various proinflammatory cytokine through indirect activation of monoamine receptors and the cAMP/PKA pathway in immune cells and/or direct effect on mast cells and other immune cells [49]. In conclusion, maprotiline is likely to reduce the proinflammatory cytokine and NO release from immune intestinal cells through this plausible mechanism.

Enhanced histamine levels are common in the mucosa and intestinal secretions of patients with IBD [50, 51]. Since maprotiline in doses that significantly inhibit the neuronal uptake of noradrenaline causes a marked inhibition of histamine [52], it may be suggested that this beneficial effect in the treatment of colitis is to some extent due to interaction with histamine.

In summary, our results confirm and extend our knowledge about anti-inflammatory effect of maprotiline on acetic acid-induced colitis in both normal and reserpinised depressed rats. Although depressive disorders are more common in such chronic disease and can exacerbate the clinical course of IBD, treatment of depression by an antidepressant drug which has beneficial effect in the course of underlying disease invokes the proverb “kill two birds with one stone.”

## Figures and Tables

**Table 1:** Effect of maprotiline (Map. 10, 20, and 40 mg/kg) on changes in the body weight in the indicated conditions.

Group	Sham	Control	Dex. 1	Map. 10	Map. 20	Map. 40

Body weight changes after four days (g)	8.8 ± 2.3	−22.5 ± 5.3^**^	0.3 ± 5.1	−13.67 ± 9.1	5.5 ± 6.5	1.0 ± 6.3

Dex. 1: dexamethasone (1 mg/kg); i.p.: intraperitoneally. Results are expressed as mean ± SEM of six rats in each group. ^**^ *P* < 0.01 compared to Sham group; one-way ANOVA followed by Tukey's test.

**Table 2:** Effect of maprotiline (Map. 10, 20, and 40 mg/kg) on changes in the body weight in the indicated conditions.

Group	Sham	Control	Dex. 1	RSP	Map. 10	Map. 20	Map. 40

Body weight changes after four days (g)	8.8 ± 2.3	−22.5 ± 5.3^**^	0.3 ± 5.1	−30.2 ± 2.8^***^	−18.7 ± 5.1^*^	−7.3 ± 6.8	−13.5 ± 3.1

Dex. 1: dexamethasone (1 mg/kg), RSP: reserpine (6 mg/kg), and i.p.: intraperitoneally. Animals were also reserpinised (6 mg/kg, i.p.) depressed. Results are expressed as mean ± SEM of six rats in each group. ^*^ *P* < 0.05, ^**^ *P* < 0.01, and ^***^ *P* < 0.001 compared to Sham group; one-way ANOVA followed by Tukey's test.

**Table 3:** Effect of maprotiline (Map. 10, 20, and 40 mg/kg, i.p.) on pathologic parameters of colitis induced by acetic acid in normal and reserpinised (6 mg/kg, i.p.) depressed rats.

Groups	Inflammation severity (0–3)	Inflammation extent (0–3)	Crypt damage (0–4)	Total colitis index (0–10)

Sham	0 (0-0)^**^	0 (0-0)^**^	0 (0-0)^**^	0 (0-0)^**^
Control	3 (2-3)	3 (2-3)	4 (1–4)	9.5 (6–10)
Dex. 1	0.5 (0-1)^**^	0 (0–2)^**^	0 (0–2)^**^	1 (0–5)^**^
Map. 10	2 (0–3)	2.5 (0–3)	3 (0–4)	7.5 (0–9)
Map. 20	1 (0-1)^*^	1 (0–2)^**^	1 (0-1)^*^	3 (0–4)^**^
Map. 40	1 (0-1)^**^	1 (0–2)^**^	0 (0-0)^**^	2 (0–3)^**^
RSP	3 (1–3)	3 (1–3)	4 (1–4)	10 (4–10)
RMAP10	2.5 (1–3)	2 (1–3)	3 (0–4)	7.5 (2–10)
RMAP20	1 (1-1)^*^	1 (0–2)^*^	1.5 (0-0)^*^	4.5 (1–3)^**^
RMAP40	1 (0–3)^**^	1 (0–3)^**^	0.5 (0-1)^*^	3 (0–7)^**^

Dex. 1: dexamethasone (1 mg/kg), RSP: reserpine (6 mg/kg), i.p.: intraperitoneally. Values are presented as median (range) of six rats in each group. ^*^ *P* < 0.05, ^**^ *P* < 0.01 compared to control; Mann-Whitney *U* test.
